# Machine Learning-Based Software Defect Prediction for Mobile Applications: A Systematic Literature Review

**DOI:** 10.3390/s22072551

**Published:** 2022-03-26

**Authors:** Manzura Jorayeva, Akhan Akbulut, Cagatay Catal, Alok Mishra

**Affiliations:** 1 Department of Computer Engineering, Istanbul Kültür University, Istanbul 34158, Turkey; 1800004575@stu.iku.edu.tr (M.J.); a.akbulut@iku.edu.tr (A.A.); 2 Department of Computer Science and Engineering, Qatar University, Doha 2713, Qatar; ccatal@qu.edu.qa; 3 Informatics and Digitalization Group, Faculty of Logistics, Molde University College-Specialized University in Logistics, 6410 Molde, Norway

**Keywords:** software defect prediction, software fault prediction, mobile application, review, systematic literature review, deep learning, machine learning

## Abstract

Software defect prediction studies aim to predict defect-prone components before the testing stage of the software development process. The main benefit of these prediction models is that more testing resources can be allocated to fault-prone modules effectively. While a few software defect prediction models have been developed for mobile applications, a systematic overview of these studies is still missing. Therefore, we carried out a Systematic Literature Review (SLR) study to evaluate how machine learning has been applied to predict faults in mobile applications. This study defined nine research questions, and 47 relevant studies were selected from scientific databases to respond to these research questions. Results show that most studies focused on Android applications (i.e., 48%), supervised machine learning has been applied in most studies (i.e., 92%), and object-oriented metrics were mainly preferred. The top five most preferred machine learning algorithms are Naïve Bayes, Support Vector Machines, Logistic Regression, Artificial Neural Networks, and Decision Trees. Researchers mostly preferred Object-Oriented metrics. Only a few studies applied deep learning algorithms including Long Short-Term Memory (LSTM), Deep Belief Networks (DBN), and Deep Neural Networks (DNN). This is the first study that systematically reviews software defect prediction research focused on mobile applications. It will pave the way for further research in mobile software fault prediction and help both researchers and practitioners in this field.

## 1. Introduction

In recent times, mobile applications play an undeniably significant role in many aspects of our lives. The new generation is growing up with new technology such as mobile phones, tablets, and laptops. We are connected by smartphones and use them for many different purposes in daily life frequently, and as such, mobile applications are becoming more and more crucial in our lives. Nowadays, we download mobile applications to access digital information, play games, learn languages, and communicate with each other. Many applications are available with free and paid versions. Compared to desktop applications, mostly mobile applications have fewer bugs/defects. Therefore, it is crucial to predict the faults before they are deployed to the mobile markets such as the Google Play Store and the iOS App Store. Currently, we download mobile applications from digital markets such as the App Store and Android Market to access information, play games, learn languages, and communicate with others. Not only are the complexities of these applications increasing dramatically, but also so are the user expectations. Many people are progressively affected by mobile application downloads, and users do not waste time using the mobile app once they observe a functional or non-functional problem [1]. Thus, it is a threat for mobile application developers and businesses to deploy software even with some minor bugs. These bugs affect the effectiveness of mobile apps and can cause unpredictable crashes [2]. When users download these problematic versions of applications, they may encounter serious problems [3]. Diagnosing mobile applications crashes can give a chance to improve faults before new releases [4]. 

A defect or fault can be an internal failure of the application and cause the system to shut down [5]. Automatic tests can identify 35% to 60% of faults, and automatic tools developed by artificial neural networks can predict 70% of faults [6]. The most used approach for software fault prediction is to analyze a set of software metrics together with labeled data with respect to different software modules and then apply machine learning (ML) methods on such datasets [7]. The aim of this prediction task is to release applications without bugs. From the 1990s until now, software defect prediction models were developed to detect faults before they are deployed to the field, and defective modules were identified before system tests by using these prediction models. Software defect prediction approaches use past software metrics and defect data to predict defective components for the new software versions. In this study, we analyzed 47 articles that focused on mobile defect prediction models and evaluated them using numerous aspects. We excluded studies that do not introduce any empirical results and are not directly related to the mobile defect prediction model development. This review identifies challenges, research gaps, and potential solutions in such a way that both researchers and practitioners can benefit.

In this study, we followed the Systematic Literature Review (SLR) methodology and responded to nine research questions defined at the beginning of this research. To the best of our knowledge, there is no other systematic review study that focuses on mobile defect prediction models and therefore, this paper provides critical insights into this field. The other sections are organized as follows: Section 2 provides the background and related work. Section 3 defines the research methodology. Results are presented in Section 4. Threats to validity are shown in Section 5. Finally, Section 6 provides the conclusions and future work.

## 2. Background and Related Work

In Section 2.1, mobile software fault prediction studies and the use of machine learning and In Section 2.2 software metrics are explained. In Section 2.3, related studies are discussed.

### 2.1. Mobile Fault Prediction and Machine Learning

Machine learning research aims to identify data patterns and discover interesting knowledge from a large amount of data. Since the 1990s, software defect prediction studies have been using machine learning algorithms to identify fault-prone classes. While software metrics are calculated based on the collected data from software repositories, fault data is retrieved from issue tracking systems. There are different software tools such as Understand to calculate software metrics automatically from software projects; however, automation of fault data collection is more challenging. Machine learning has been applied for both predicting the number of faults (i.e., a regression task) and categorization of modules into fault-prone and non-fault-prone classes (i.e., binary class classification). In machine learning, there are four learning types: supervised, unsupervised, semi-supervised, and reinforcement learning. In supervised learning, labeled data are needed to build the models. In unsupervised learning, hidden structures in data are discovered by detecting the feature correlations. Clustering and dimensionality reduction algorithms are considered under the unsupervised learning category. Semi-supervised learning is used when there are very limited fault data (e.g., 5–15%). The last category is reinforcement learning that uses software agents to learn the environment by using a trial-and-error basis and also, applies feedback mechanism.

### 2.2. Software Metrics

The motivation for monitoring and analyzing software metrics is that they are commonly used to determine the quality of software components and/or products and to take actions to improve their quality. In the early 1990s, object-oriented metrics were proposed and used to characterize software components. Researchers such as Chidamber and Kemerer, Li, Lorenz and Kidd have defined different metrics to measure complexity and calculate the static aspects of software design. The most well-known among them are; Number of Children (NOC), Depth of Inheritance Tree (DIT), Coupling Between Objects (CBO), Weighted Methods per Class (WMC), Lack of Cohesion in Methods (LCOM), and Response for a Class (RFC), Number of Ancestor Classes (NAC), Class Method Complexity (CMC), Number of Local Methods (NLM), Number of Descendent Classes (NDC), Coupling Through Message Passing (CTM), and Coupling Through Abstract Data Type (CTA), Number of Public Methods (NPM), Number of Methods (NM), Number of Public Variables (NPV), Number of Variables per Class (NV) [8]. In addition to these code metrics, project managers also track developer productivity, process, operational, test, and customer satisfaction metrics as well.

### 2.3. Related Work

In this sub-section, we discuss the previously published review papers on defect prediction. Catal and Diri analyzed software defect prediction articles with respect to different software metrics, datasets, and approaches [9]. Malhotra and Jain analyzed the prior publications and published a review paper on defect prediction [10]. Malhotra reviewed publications from 1991 to 2013 that apply machine learning methods for software defect prediction [11]. Radjenovic et al. analyzed defect prediction papers published from 1991s to 2011 and reported that machine learning methods and object-oriented metrics were widely applied for fault detection in the literature [12]. Misirli et al. analyzed 38 publications using machine learning methods and presented a systematic mapping study. They reported that machine learning algorithms such as Bayesian networks were used in 70% of studies [13]. Morera et al. reviewed studies on software defect prediction from 2002 to 2014, selected 40 studies, and presented a systematic mapping study on software defect prediction. They discussed the performance of machine learning methods such as Random Forest, Naïve Bayes, Logistic Regression, and Decision Trees [14]. Ozakıncı et al. reviewed publications published between 2000 and 2016 and selected 52 publications. They investigated the aim, development, progress, advantages, and components of models and presented a systematic review [15]. Son et al. performed a systematic mapping study of software defect prediction studies using 156 articles and reported that very few studies described cross-project defect prediction [16].

In addition, several systematic literature reviews (SLR) and systematic mapping studies (SMS) have been published in the software engineering discipline so far. Najm et al. analyzed studies published until 2017, which used a Decision Trees (DT) algorithm for software development effort estimation in their SMS study. The selected publications are categorized based on publication platform, analysis model, research strategy approaches applied in organizations [17]. Alsolai et al. analyzed publications related to the software maintainability prediction and presented an SLR study. They reported that the authors used some private datasets in some papers, evaluated their models using k-fold cross-validation approaches, and applied named regression algorithms [18]. Auch et al. analyzed studies published between 2002s and 2019, focused on similarity-based analyses on software applications, and selected 136 articles. They applied inclusion and exclusion criteria to select related studies, identified the applications’ similarities, and presented a systematic literature review on similarity analysis of software applications [19]. Degu analyzed 31 studies related to the Android application memory and energy performance and published an SLR paper. This study presented a review to classify the research results covering Android application memory and energy work, resource leaks, and performance testing approaches and threats [20]. Kaur and Kaur presented an SLR study on the mobile application development and testing effort estimation. They analyzed and correlated existing test evaluation methods for conventional mobile and desktop applications [21]. Del Carpio and Angarita published an SLR study to investigate the trends in software engineering processes using deep learning. They stated that deep learning methods such as Convolutional Neural Networks (CNN), Long-Short Term Memory (LSTM), and Recurrent Neural Networks (RNN) are used for fault detection, analyzing images, demands, and diagnose errors on the monitoring stage. In addition, they identified the usage of deep learning for defect prediction, classification problems, visualization, test, and analysis software requirements [22]. Kaur presented an SLR study on code smells and quality attributes relations. They reported that various code smells could have differing results against other software quality attributes [23].

We also observed studies that evaluated the effect of data sampling for defect prediction because the datasets in software defect prediction are mostly imbalanced and therefore, data sampling algorithms are needed. For example, Kaya et al. developed defect prediction models using machine learning algorithms, data sampling approaches, and design-level metrics. They reported that data sampling approaches help to improve the performance of models. They stated that the Adaboost ensemble algorithm provides the best performance for defect prediction [24].

Additionally, some papers presented novel models based on mobile application datasets. For example, Kaur et al. analyzed process metrics to predict defects of mobile applications and performed experiments using publicly available mobile applications datasets. They focused on regression algorithms and applied process and code metrics for model development. A process metrics-based machine learning model provided the best performance according to their experiments [25]. Zhao et al. presented a deep learning-based model for just-in-time defect prediction of Android applications. They applied their proposed model on 12 applications datasets and stated that the novel Imbalanced Deep Learning (IDL) model provided the best performance among others [26].

Additionally, some studies focused on the hyper-parameter tuning for improving the performance of models. For example, Sewak et al. analyzed different types of LSTM architectures for Intrusion Detection Systems and demonstrated the benefits of hyper-parameter tuning in LSTM models [27]. Software defect prediction can be used in many of the fields of engineering described [28] and it can be used to compare Machine Learning and Statistical methods for classification fault and non-fault classes. Internet of Things (IOT) was used to automate applications for our needs. Bhana et al. reported real-time applications of defect prediction that use restored data in the cloud. Their model can be implemented in daily life using a real-time application [29]. Pandey et al. performed a model using Long Short-Term Memory for cross-project defect prediction. They experimented with 44 projects with imbalanced datasets and compared DNNAttention with unsupervised learning [30].

## 3. Research Methodology

**Figure 1:**
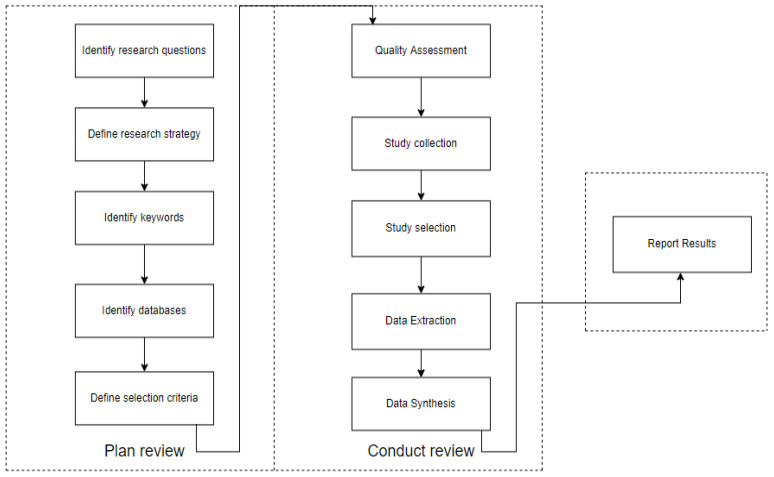
Systematic Literature Review process.

This section presents the research methodology. This study followed a similar methodology proposed by [31]. We organized the systematic review process shown in Figure 1 and followed steps to reduce risk bias in the study. First, research questions were identified, and papers were retrieved from scientific databases. Study selection criteria were applied to the papers, and a subgroup was selected for the quality assessment step. Each paper was scored based on eight quality assessment questions shown. Data were extracted and synthesized then the final subgroup of studies (i.e., 47 papers) were selected to respond to the research questions. All of these 47 papers were read in full and research questions were answered. Research questions of this study are presented in Table 1.

**Figure 2:**
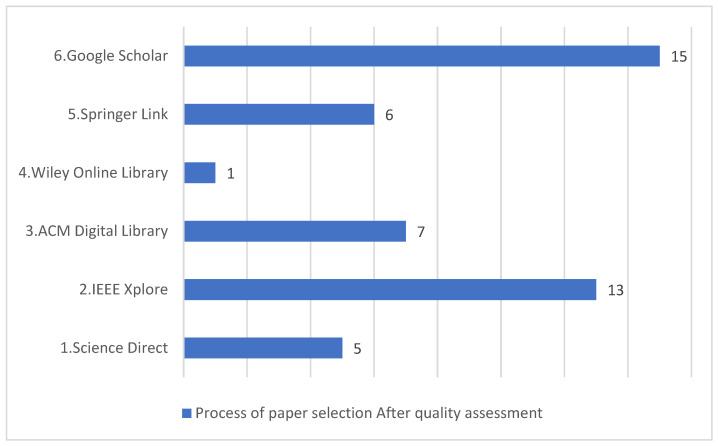
Distribution of the selected papers.

The following databases were used to retrieve relevant papers: IEEE Xplore, Science Direct, ACM Digital Library, Wiley Online Library, Springer Link, and Google Scholar. The search spanned the last 10 years to identify up-to-date papers. Table 2 shows the exclusion criteria used in this study. The following search criteria were applied: ((“machine learning” OR “artificial intelligence”) AND “mobile software” AND (“fault prediction” OR “defect prediction” OR “software quality”)). Figure 2 shows the distribution of papers per database and the number of papers at each stage (i.e., after initial query, after exclusion criteria, and after quality assessment).

Figure 2 shows that most papers were retrieved from the IEEE Xplore database and Google Scholar also included a similar number of papers in the final selection.

**Figure 3:**
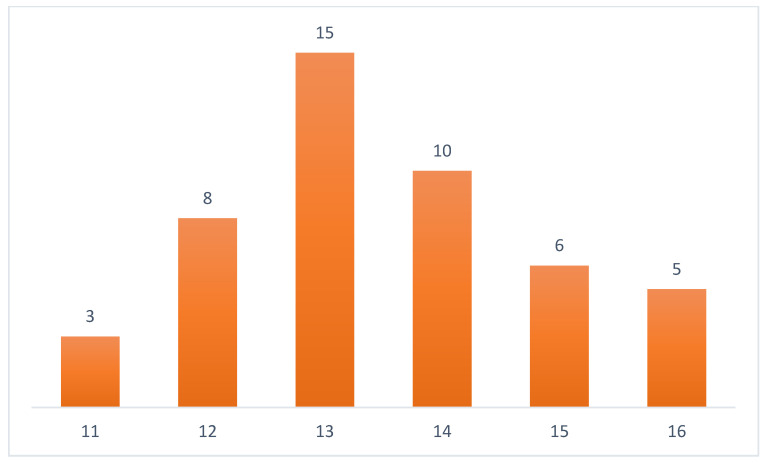
Quality score distribution of selected papers (x axis: paper score, y-axis: number of papers).

After the exclusion criteria were applied, we graded papers for quality assessment using the approach proposed [33]. Table 3 shows quality evaluation questions. Papers with scores lower than 10 were excluded from the list. Figure 3 shows the quality distribution of papers. If the answer is “yes” for the question, the paper receives two points, the “partial” response receives one point, and “no” answer receives no points.

**Figure 4:**
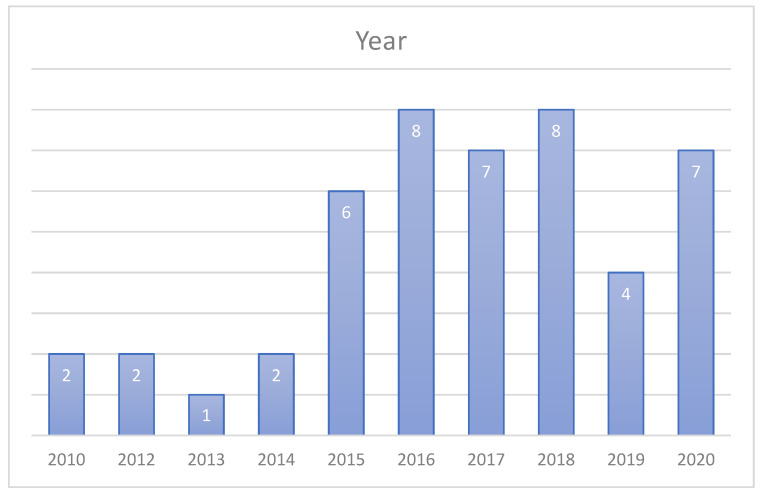
Selected publications per year.

After quality assessment questions were applied, publications were synthesized. In Figure 4, the distribution of the selected publications per year is shown. As shown in this figure, within the last five years, more papers were published on this topic and this field is still active.

**Figure 5:**
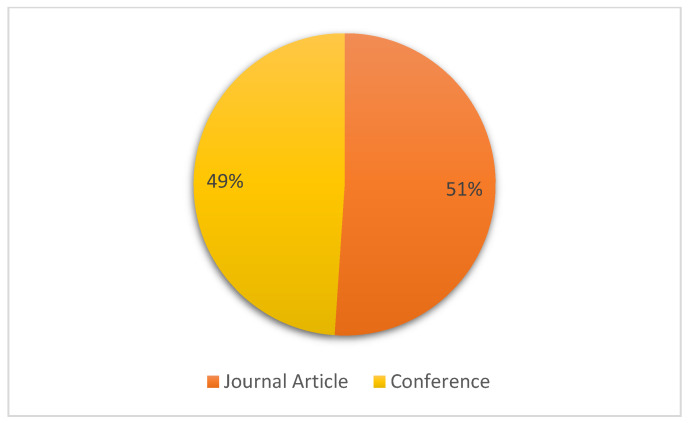
Distribution of type of publications.

Figure 5 presents the representation of the type of publications. Nearly half of the papers are journal papers, and the rest are conference proceedings. This indicates that some researchers prefer publishing this type of paper in conferences; however, a sufficient number of journal articles are evaluated in this SLR paper.

## 4. Results

In this section, we explain our responses to each research question.

### 4.1. RQ-1: Platforms

This section provides the details of the platforms used in primary studies. As shown in Table 4, the most used platform is the Android platform, which has 21 publications. Windows Mobile platform is used only in one publication. Web applications were used in 20 publications and Mobile Applications used in five publications. This shows that most researchers prefer the Android platform for defect prediction studies. The main reason might be the open source nature of the Android platform and the applications released in this platform. There were also plenty of web applications used in these papers.

### 4.2. RQ-2: Datasets

The datasets related to software defect prediction studies are available in repositories. Table 5 presents the repositories, datasets, and web addresses.

**Figure 6:**
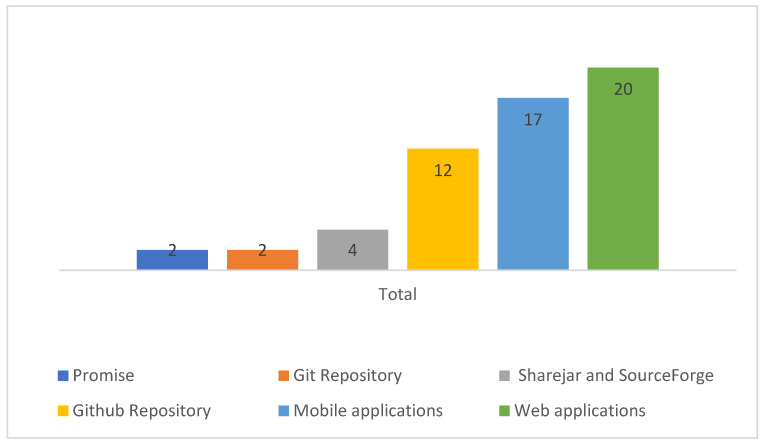
Repositories.

Figure 6 shows the distribution of repositories. As shown in the figure, most researchers preferred Github repository to host their datasets and other repositories such as SourceForge are not widely preferred.

### 4.3. RQ-3: Machine Learning Types

**Figure 7:**
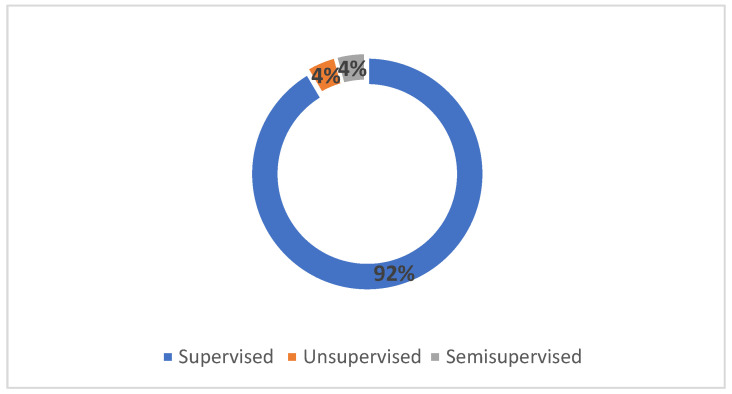
Distribution of Machine Learning Types.

Supervised learning algorithms were preferred in 43 papers. The other machine learning types (i.e., unsupervised and semi-supervised) were used in four publications (two papers per each type). Figure 7 shows the distribution of ML types used in selected publications. This indicates that most of the researchers preferred supervised learning approaches when developing models for mobile applications. However, the literature also includes unsupervised fault prediction models [34] and semi-supervised fault learning models [35]. Additionally, noisy instances can be removed from the datasets to improve the overall performance of the supervised models [36].

### 4.4. RQ-4: Machine Learning Algorithms

In studies that do not employ deep learning techniques, for the most part, a static feature selection that is manually chosen by knowledgeable domain experts is preferred. However, we also observed that the Correlation-based Feature Selection (CFS) method was used in several studies [37,38,39,40,41] as the feature subset selection technique. Secondly, gain ratio attribute evaluation is used [42,43,44] to reduce the high-dimensionality and further improve efficiency. Alternatively, machine learning models such as Logistic Regression (LR) and Random Forest (RF) were built [45,46] for the same purpose. The different methods were noted as applying evolutionary techniques [47], statistical feature selection [48], Principal Component Analysis [49], and T-test analysis-based feature selection [50].

**Figure 8:**
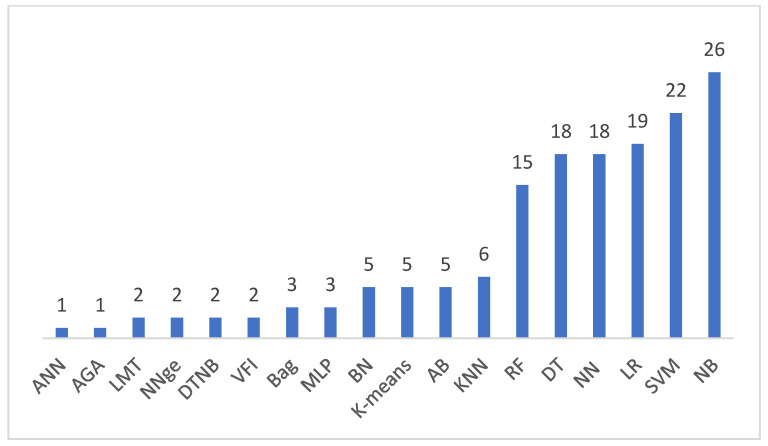
Machine Learning Algorithms.

In selected studies, eighteen machine learning methods were used. The algorithms are as follows: Naïve Bayes (NB) in 26 studies, Support Vector Machines (SVM) in 22 studies, Logistic Regression (LR) were in 19 studies, Neural Network (NN) in 18 studies, Decision Tree (DT) in 18 studies, Random Forest (RF) in 15 studies, K-nearest Neighbors (KNN) in six studies, Alpha-beta pruning (AB), K-means, and Bayesian Network (BN) in five studies, respectively, Bootstrap aggregating (Bag) and Multilayer Perceptron (MLP) in three studies, respectively, and Voting Future Intervals (VFI), DTNB, Non-Nested Generalization (NNge) and Logistic model tree (LMT) were used in two studies, respectively. Artificial Neural Network (ANN), and adaptive genetic algorithm (AGA) were used only in one study each. Figure 8 shows the distribution of algorithms. Based on this analysis, we can state that the top three applied algorithms are Naïve Bayes, Support Vector Machines, and Logistic Regression algorithms.

### 4.5. RQ-5: Evaluation Metrics

**Figure 9:**
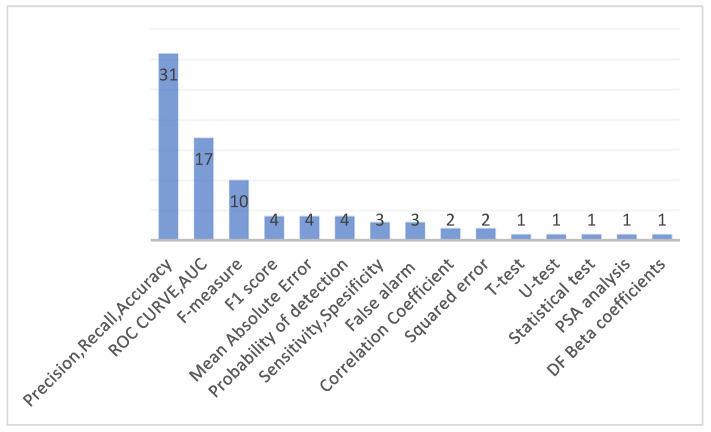
Evaluation metrics.

We identified 19 evaluation metrics in the selected articles. The Precision, Recall, and Accuracy metrics set was used in 31 articles. Seventeen articles used ROC Curve and Area under ROC curve (AUC) metrics. F-measure was used in 10 publications. F1 score was used in four publications. Mean Absolute Error was used in four publications. The distribution of evaluation metrics is presented in Figure 9. This figure indicates that most researchers preferred the precision and recall parameters while evaluating their models. Additionally, AUC is widely used by researchers in this field.

### 4.6. RQ-6: Validation Approaches

**Figure 10:**
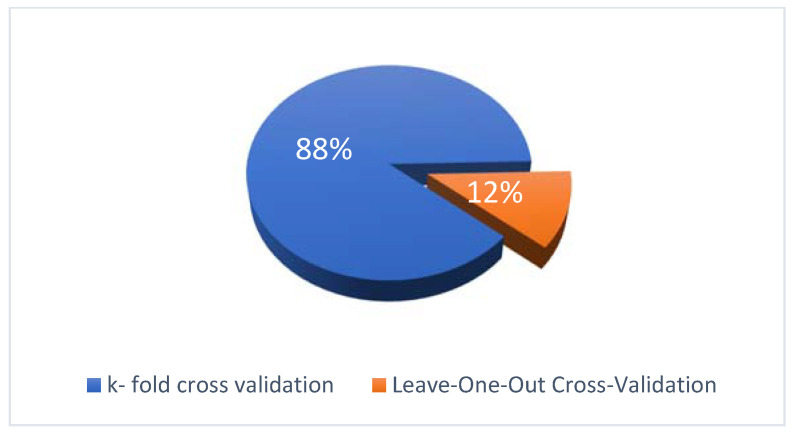
Distribution of validation approaches.

Eighty-eight percent of studies used k-fold cross-validation in these papers. Leave-one-out validation was applied in 12% of studies. Figure 10 represents the distribution of applied validation approaches. This figure indicates that most researchers prefer the use of K-fold cross-validation in mobile defect prediction.

### 4.7. RQ-7: Software Metrics

**Figure 11:**
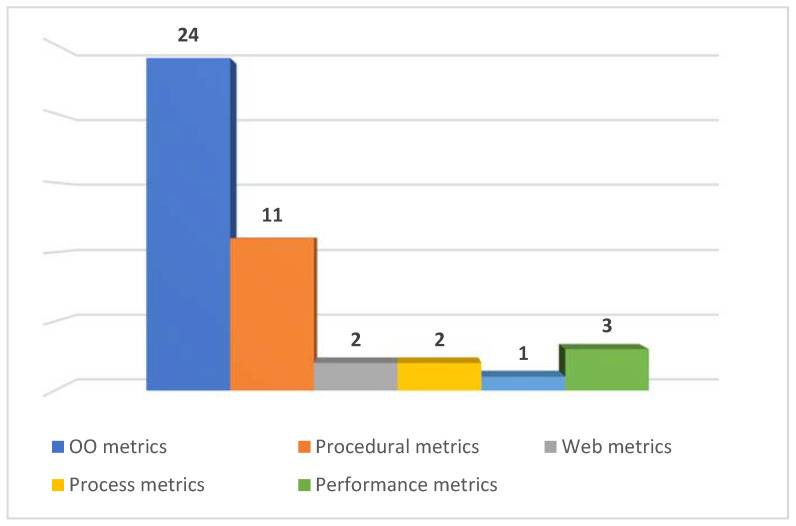
Distribution of metrics types.

We extracted all the metrics used in primary studies. As seen in selected publications, many metric types have been used. Therefore, we decided to categorize metrics. Object-oriented metrics were used in 24 publications. Procedural metrics were used in 11 publications. Web metrics were used in two publications. Process metrics were applied in two publications. Performance metrics were used in two publications. Figure 11 shows the distribution of metric types applied in selected papers. This figure indicates that most researchers preferred object-oriented metrics in mobile defect prediction studies.

### 4.8. RQ-8: The Best Algorithm

**Figure 12:**
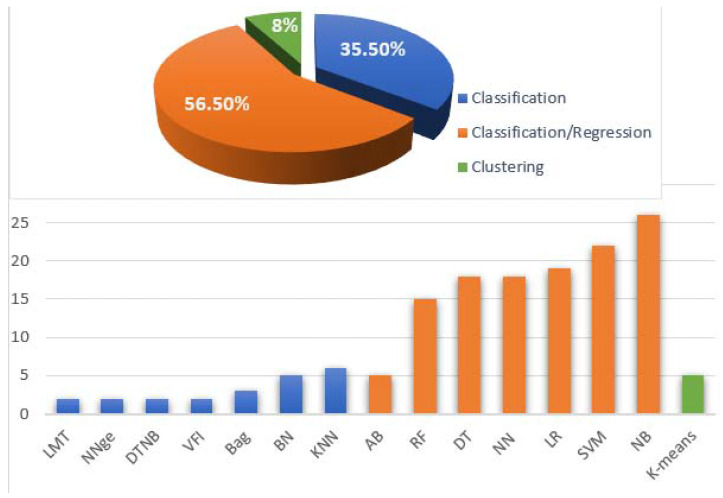
Machine Learning Algorithms Performance.

We categorized algorithms into two categories: traditional machine learning algorithms and Deep Learning algorithms. Figure 12 shows the best performing machine learning algorithms. Support Vector Machines (SVM) was identified four times as the best algorithm in publications. Random Forest (RF) and Multilayer Perceptron (MLP) were reported the best algorithm in three publications. Bayesian Network (BN), Naïve Bayes (NB), and Logistic Regression (LR) were chosen twice as the best algorithm. The other algorithms, Multiple Linear Regression (MLR), Multinominal Naïve Bayes (MNB), Co-trained Random Forest (Co Forest), Adaptive Boosting (AdaBoost), Gradient Boosting (GBDT), J48, Bootstrap aggregating (Bag), K-means++ (K-means clustering), KNN (K-nearest neighbors), Artificial Neural Network (ANN), An adaptive genetic algorithm (AGA), were reported only once as the best algorithm. We observed that ensemble techniques, namely Random Forest, Bootstrap Aggregating, Adaptive Boosting, and Gradient Boosting are also used.

**Figure 13:**
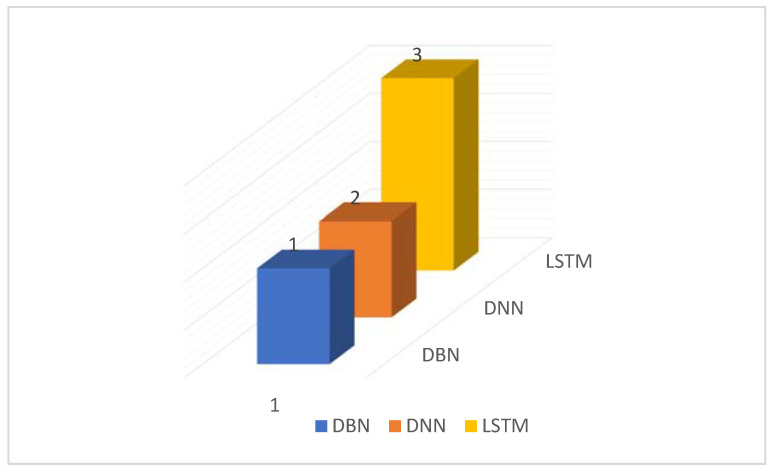
Distribution of Deep Learning Algorithms.

Figure 13 shows the distribution of Deep Learning algorithms. Long Short-Term Memory (LSTM) was specified in two publications as the best algorithm. Based on this analysis, we can state that SVM, MLP, and RF are the top three shallow learning algorithms in terms of performance and LSTM is the most important deep learning algorithm among other deep learning algorithms.

### 4.9. RQ-9: Challenges

In this section, we present the main challenges and proposed solutions reported in mobile defect prediction studies. Table 6 shows the main challenges and possible solutions with the references.

## 5. Discussion

In this section, we present the general discussion and validity considerations of this systematic literature review.

### 5.1. General Discussion

This study aims to collect, synthesize, and evaluate mobile application defect prediction publications using machine learning techniques. To the best of our knowledge, there has been no similar SLR paper published on this topic yet. Therefore, we performed this SLR study and aimed to answer some research questions that we defined at the beginning of this research. We believe that the observations and suggestions will pave the way for further research and help both practitioners and researchers in this field. Responses to research questions are briefly discussed as follows:

RQ1—We noticed that most papers addressed the Android platform but the Windows mobile operating system was discussed in only 2% of the studies. We also did not see any paper that focused on the iOS platform. The reason is probably related to the open source code bases of Android-based applications, which supported the researchers in a way that they were able to calculate the software metrics and collect the defect information from different publicly available repositories. Since many mobile platforms use the Android operating system, many researchers prefer to perform experiments on this platform.

RQ2—Many datasets have been stored in Github or git repositories for defect prediction. These repositories are widely used by practitioners and researchers, therefore, the available datasets are mostly located in these platforms. This is also related to the open source nature of Android applications; they are mostly hosted in these platforms. There were also a few datasets that used other platforms, however, their percentage was lower compared to the use of Github-related repositories.

RQ3—Most of the studies used supervised learning approaches; they were limited number of papers that used unsupervised and semi-supervised learning techniques. The reason is that most researchers were probably able to obtain the available defect information from the publicly available repositories and therefore, they aimed to build supervised learning models instead of unsupervised or semi-supervised learning models. However, it is also possible to carry out some experiments in the context of available defect information by simulating different scenarios. There is still some room for further research on the use of these less preferred machine learning types.

RQ4—Naïve Bayes (NB), Support Vector Machines (SVM), and Logistic Regression algorithms are the most preferred algorithms. The reason is most probably that researchers preferred the widely used machine learning algorithms such as SVM and NB in their experiments. Previously, it has been also demonstrated that NB provides high performance in software defect prediction [51], and therefore, it might have been preferred in the mobile application defect prediction studies as well.

RQ5—Most of the papers used Precision, Recall, and Accuracy metrics to evaluate the performance of the models and also the Area Under ROC curve (AUC) metric was preferred by researchers. These metrics are widely used in machine learning studies and therefore, researchers probably selected these metrics. Accuracy is not a good metric for defect prediction studies because these datasets are imbalanced and the accuracy metric cannot be used alone to judge the performance of the models; it must be used together with other metrics such as precision and recall.

RQ6—Most studies used the k-fold cross-validation strategy for the evaluation of the model performance. This is also the widely used evaluation approach among machine learning approaches and therefore, researchers might have preferred to use this strategy. There are also other alternatives such as leave-one out technique; however, k-fold cross-validation was applied by most researchers. There is also possibility to perform k-fold n times, which can be called k*n cross-validation; however, the use of this strategy in these papers was quite limited, although this approach can present more statistically sound results.

RQ7—Most of the studies used object-oriented metrics. This is probably due to the widespread adoption of object-oriented programming paradigms in the software industry. However, new metrics can be proposed and evaluated by researchers for mobile applications. This might be a potential research topic for researchers.

RQ8—Support Vector Machines (SVM), Random Forest (RF), Multilayer Perceptron (MLP) were among the best performing algorithms. Among deep learning algorithms, LSTM provided the best performance. Since the training of deep learning models requires more time and data, in some cases, researchers and practitioners can consider the scale of the dataset before building the prediction model. If traditional machine learning algorithms (i.e., shallow learning) can provide high performance, more complex algorithms such as deep learning might not be needed.

RQ9—Several challenges were mentioned to answer this research question. We extracted these challenges from the papers if they were mentioned. However, there is a possibility that authors might not have discussed the challenges in the paper explicitly. In such cases, we were unable to add those challenges. If the challenge has not been experienced by researchers and mentioned as future work, they were also not included. There might be additional challenges that are missing in this paper; however, we aimed to collect them from the available literature.

### 5.2. Threats to Validity

We selected publications from six digital platforms using our search criteria and also conducted a snowballing process. Authors held several meetings to minimize the researcher bias. However, there might be some papers in some electronic databases that we have missed in this research. Additionally, new papers are also published very frequently and therefore, we might have missed some new papers published recently. Another threat is the use of the search criteria. There might be more synonyms that could have been used in this research and we have missed some papers due to this issue.

## 6. Conclusions and Future Work

This study presented the results of a systematic literature review on mobile fault prediction using machine learning. A total of 721 publications were retrieved from electronic databases, and after study selection criteria, 47 publications were selected. The selected publication is classified based on platforms, datasets, machine learning types, machine learning algorithms, evaluation metrics, validation approaches, software metrics, best machine learning and deep learning algorithms, challenges and gaps, and the corresponding results are reported. The Android platform was mostly preferred by researchers. Furthermore, there exists a limited number of repositories and datasets for mobile defect prediction studies. Most researchers used object-oriented metrics in mobile defect prediction. Most of the studies used supervised learning algorithms instead of unsupervised and semi-supervised learning algorithms. This means that there is still a potential for further research using unsupervised and semi-supervised learning for mobile defect prediction. We are planning to build novel prediction models using these algorithms for the Android platform.

## Figures and Tables

**Table 1:** Research questions in this research.

RQ	Research Questions
RQ1	Which platforms are addressed in mobile defect prediction?
RQ2	Which datasets are used in mobile defect prediction studies?
RQ3	Which machine learning types are used in mobile defect prediction studies?
RQ4	Which machine learning algorithms are applied in mobile defect prediction?
RQ5	Which evaluation metrics are used in mobile defect prediction?
RQ6	Which validation approaches were used in mobile defect prediction?
RQ7	Which software metrics were adopted in mobile defect prediction?
RQ8	Which ML algorithm works best for mobile defect prediction?
RQ9	What are the challenges and research gaps in mobile defect prediction?

**Table 2:** Exclusion criteria [32].

ID	Exclusion Criteria
1.	The paper includes only an abstract (this criterion is not about the accessibility of the paper, we included both open access and subscription basis papers)
2.	The paper is not written in English
3.	The article is not a primary study paper
4.	The content does not provide any experimental results
5.	The study does not describe in detail how machine learning is applied

**Table 3:** Quality evaluation questions. “Yes” scores 2; “partial” scores 1; “no” scores 0.

ID	Questions
Q1	Are the aims of the study clearly declared?
Q2	Are the scope and context of the study clearly defined?
Q3	Is the proposed solution clearly explained and validated by an empirical study?
Q4	Are the variables used in the study likely to be valid and reliable?
Q5	Is the research process documented adequately?
Q6	Are all study questions answered?
Q7	Are the negative findings presented?
Q8	Are the main findings stated clearly in terms of credibility, validity, and reliability?

**Table 4:** Platforms.

Platforms	Total
Android	21
Windows Phone	1
Web Applications	20
Mobile Applications	5

**Table 5:** Repositories.

Repositories	Datasets	Web Address
GIT Repository	Contact, MMS, Bluetooth, Email,	https://android.googlesource.com (accessed on 15 June 2021)
Sharejar and Source Forge	Calendar, Gallery2, and Telephony	https://sourceforge.net/p/bitweaver/bugs/ http://securityfocus.com https://sourceforge.net/p/Webcalendar/bugs
GitHub Repository	Bootstrap, Avaya Communicator, The K-9 Mail client, Space Blaster game, K-9 issue report.	https://github.com/bpellin/keepassdroid https://github.com/adrian/upm-android http://android.scap.org.cn/ http://cve.mitre.org/ https://github.com/breezedong/DNN-based SFP https://github.com/JesusFreke/smali/wiki https://github.com/tapjdey/release_qual_model https://github.com/geometer/FBReaderJ
Mobile Applications Web Applications	Connectbot, Boardgame Geek, AnkiDroid, Android Wallpaper, Quiksearchbox, Afwall, Alfresco, AndroidSync F-droid Fusion Android PHP web apps Drupal project Tech-terms	https://techterms.com/definition/repository http://pallergabor.uw.hu/androidblog/dalvik_opcodes.html https://source.android.com/devices/tech/dalvik/dalvikbytecode http://searchoracle.techtarget.com/definition/repository http://code.google.com/p/sipdroid/ https://forum.xda-developers.com/showthread.php?t=1800090 https://tinyurl.com/m722ouohttps://tinyurl.com/ya533yya https://tinyurl.com/y9vcudjthttps:/Iplay.google.comlstore/search?q=weather%20forecast http://f-droid.org! http://23.92.18.210:8080/FusionWeb/viewer.jsp http://sharlwinkhin.com/phpminer.html http://www.drupal.org/ https://techterms.com/definition/repository

**Table 6:** Challenges and possible solutions.

Challenges	Proposed Solutions	Reference
Metric selection limitations for mobile software	Use alternate code and process metrics	[3,18]
Faults in Android data	Remove faults	[9]
Limited mobile app repository	Use of public repository	[11,27,28,36]
Repeated data/code in the project	Domain Adaptation	[26]
Small dataset problem	Not mentioned	[22]
Different programming language problem	Defect prediction only GIT open-source Android, Java, and C++ uncertain	[8,10,26]
Modeling problem	Not mentioned	[4,11,30]
Different platforms and languages	Not mentioned	[18,21]
Extensive datasets	Not mentioned	[16]
Not fully automated	Manually code, log, bug, and review control	[11]
Imbalance Class problem	Sampling methods, Under sampling methods	[7,12,22]
Manual feature engineering	Not mentioned	[26]

## Data Availability

Not applicable.
